# Two Faces of TGF-Beta1 in Breast Cancer

**DOI:** 10.1155/2014/141747

**Published:** 2014-05-07

**Authors:** Joanna Magdalena Zarzynska

**Affiliations:** Department of Food Hygiene and Public Health, Faculty of Veterinary Medicine, WULS-SGGW, Nowoursynowska 159, 02-776 Warsaw, Poland

## Abstract

Breast cancer (BC) is potentially life-threatening malignancy that still causes high mortality among women. Scientific research in this field is focused on deeper understanding of pathogenesis and progressing of BC, in order to develop relevant diagnosis and improve therapeutic treatment. Multifunctional cytokine TGF-**β**1 is one of many factors that have a direct influence on BC pathophysiology. Expression of TGF-**β**1, induction of canonical and noncanonical signaling pathways, and mutations in genes encoding TGF-**β**1 and its receptors are correlated with oncogenic activity of this cytokine. In early stages of BC this cytokine inhibits epithelial cell cycle progression and promotes apoptosis, showing tumor suppressive effects. However, in late stages, TGF-**β**1 is linked with increased tumor progression, higher cell motility, cancer invasiveness, and metastasis. It is also involved in cancer microenvironment modification and promotion of epithelial to mesenchymal transition (EMT). This review summarizes the current knowledge on the phenomenon called “TGF-**β**1 paradox”, showing that better understanding of TGF-**β**1 functions can be a step towards development of new therapeutic approaches. According to current knowledge several drugs against TGF-**β**1 have been developed and are either in nonclinical or in early stages of clinical investigation.

## 1. Introduction

Breast cancer (BC) is the most common and fatal cancer worldwide, with high morbidity and mortality in woman. It is ranked on the second place in mortality among cancer types [1], causing death of about 350,000 women in both developed and developing countries every year [2]. More than 90% of lethality in cancer patients is caused by metastasis, and the occurrence of distant metastases severely limits the prognosis of breast cancer patients [3]. The 5-year survival rate for patients with breast cancer drops precipitously from 98% for individuals with localized disease to 23% for those with metastatic disease [4]. Many factors are involved in the pathogenesis and progression of BC, including genetic, biological, and environmental factors, as well as lifestyle [2]. Cytokines belong to the biological factors, playing pivotal role in modulation of cellular growth, maturation, differentiation, and cancer progression. One of the cytokines responsible for regulation of cell behavior is Transforming Growth Factor-*β* (TGF-*β*), which has been extensively studied in regard to its various effects exerted on epithelial cells and derivative carcinoma cell populations* in vitro* and* in vivo*. TGF-*β* has been shown to inhibit epithelial cell cycle progression and promote apoptosis. These effects together significantly contribute to the tumor suppressive role of TGF-*β* during carcinoma initiation and progression. TGF-*β* is also able to promote epithelial to mesenchymal transition (EMT), in order to modulate immune system and tumor microenvironment, which have been associated with increased tumor cell motility, invasion, and metastasis [5–8]. In several types of human carcinomas, mutations or loss of heterozygosity (LOH) in central components of the TGF-*β* pathway has been observed [9, 10]. Functional insights into TGF-*β* pathway are vital for developing new therapeutic approaches in cancer. This publication is focused on the influence of TGF-*β* on human breast cancer pathophysiology.

## 2. TGF-***β*** Characteristics

The superfamily of TGF-*β* cytokines consists of over 40 proteins, including: TGF-beta (*β*), activins (A, AB, B, C, E), inhibins (A, B), bone morphogenetic proteins (BMPs), and growth/differentiation factors (GDFs) [11, 12]. TGF-*β* is a polypeptide (112 AA), encoded by a gene located on the long arm of chromosome 19 (19q13) in humans [13]. TGF-*β* occurs in five isomeric forms (60–80% of homology), from *β*1 to *β*5, produced by alternative splicing. TGF-*β* 1–3 are found in humans, other mammals, and birds [12]. TGF-*β*1 activity is pleiotropic. It is a predominant isoform in humans, synthesized by almost all cells, primarily by platelets, Treg cells, macrophages/monocytes, lymphocytes, fibroblasts, epithelial cells, and dendritic cells [12]. TGF-*β*1 plays pivotal roles in modulation of cellular growth, maturation and differentiation, ECM (extracellular matrix) formation, homeostasis, endothelial cell plasticity, immunoregulation, apoptosis, angiogenesis, and cancer progression [14–19]. TGF-*β*1, -2, and -3-specific mRNAs are detected in majority of primary breast cancers [20]. Plasma levels of TGF-*β* have also been reported to be elevated in patients with breast cancer. These levels correlate with disease stage and decrease following resection of primary tumor [20]. The members of TGF-*β* family are predictors of poor response to chemotherapy in women with BC [21].

TGF-*β*1 occurs as a homodimer (25 kDa molecular weight) and is released from cells as an inactive precursor, with propeptide latency-associated protein (LAP). TGF-*β*1 is connected by a disulphide bond through the LAP with Latent TGF*β* binding protein (LTBP). LTBP1-4 is a component of the ECM and is necessary both for synthesis of TGF-*β*1 and its storage [12]. Thrombospondin-1 (TSP-1), an ECM protein, allows releasing TGF-*β*1 in an active form by changing the conformation of LTBP. Increased TGF-*β*1 activity has been observed in response to angiotensin II, low density lipoproteins (LDL), glucose, thromboxane A2, integrin, calpain, cathepsin D, chymase, elastase, endoglycosidase F, kallikrein, MMP-9, neuraminidase, plasmin, and TSP-1. Among the known inhibitors of this cytokine are follistatin, decorin, and *α*2-macroglobulin [5, 12].

Studies carried out on the mouse model indicate a key role of the members of TGF-beta family (TGF-*β*1, -*β*2, -*β*3) in establishing proper mammary gland architecture, regulating stem cell kinetics, maintaining the mammary epithelium in a functionally undifferentiated state, and inducing apoptosis in the involuting gland [11]. TGF-*β*1 inhibits mammary epithelial cells (MECs) proliferation in an auto/paracrine manner [11]. It is expressed during all stages of mammary gland development, with the lowest expression level during lactation. Studies on rodents and human mammary carcinoma cells implicate that prolactin, growth hormone (GH), EGF, and IGF-I act as inhibitors of TGF-*β*1 expression in MECs, while somatostatin and sex steroids are shown to stimulate the expression. Expression of TGF-*β*1 is also negatively regulated by ECM. Transcription of TGF-*β*1 is high in the absence of ECM and is considerably lower in the presence of endogenously synthesized basement membrane [22].

## 3. TGF-***β***1 Signaling

The TGF-*β*1 signaling pathway depends on the tissue context [23]. Specific membrane binding receptors are needed for signaling activity of TGF-*β*1 in the cells. The best known are dimeric proteins, T*β*RI (53 kDa), T*β*RII (75 kDa), and T*β*RIII (280 kDa). T*β*RI (also known as activin receptor-like kinase 5, ALK5) and T*β*RII are transmembrane receptors, which have serine-threonine kinase activity of the intracellular domains. The extracellular part of T*β*RII is activating the intracellular part by binding the ligand (autophosphorylation). Then the complex joins and recruits T*β*RI, which determines specificity of TGF*β* recognition. Activated T*β*RII kinase phosphorylates serine fragments of sequence TTSGSGSG in GS domain (domain rich in Gly and Ser) of T*β*RI, thus leading to activation of serine-threonine kinase in the receptor, and thereby the signal transduction cascade inside the cell is initiated [5, 8, 12, 23, 24]. The full heterodimeric complex is needed for correct signal transduction. Without presence of T*β*RI TGF-*β*1 can bind to the T*β*RII, but the transduction does not occur. In the absence of T*β*RII, the cells are insensitive to the action of TGF-*β*. T*β*RIII has a structure of betaglycan and has no enzymatic activity [12]. It is a coreceptor presenting TGF-*β* to the other receptors. T*β*RIII may also be an inhibitor of the signal transduction by preventing TGF-*β*1 binding to T*β*RII and T*β*RI in the mechanism independent of ligand binding, so in such situations it exerts regulatory function [5, 12, 23, 25].

Further signal transduction to the nucleus occurs with participation of cytoplasmic proteins, which are transcription factors and intracellular transmitters from Smad family. After activation of T*β*RI, the signal activates Smad2 and Smad3 proteins (R-Smad subclass; receptor regulated Smad) bound to the receptors, by phosphorylation of their C-terminal (SXS motif) residues. Phosphorylated R-Smad can be separated from the connection with the receptor and from protein SARA (Smad Anchor for Receptor Activation). SARA is a cytoplasmic protein anchored in the cell membrane that binds both the R-Smad and heteromeric complex of TGF*β*1 receptors. It recognizes the nonphosphorylated R-Smad, joins it to the complex, and then dissociates. The next step is formation of a functional trimeric complex by phosphorylated R-Smad and co-Smad (common partner Smad), namely Smad4, and then this complex is translocated to the nucleus, where it regulates the transcription of TGF-*β*1-dependent genes, thus Smads have the activity of transcription factors [5, 24, 26, 27]. Smad4 cooperates with other transcription factors, such as FoxH1, Mixer, Runx-related proteins, and E2F, as well as transcriptional coactivators (e.g., p300 and CBP) and corepressors (e.g., SKI and SnoN, prooncoproteins) in the regulation of target genes [5, 26].

The activity of TGF-*β* signaling pathway is regulated by a negative regulatory feedback loop mediated by I-Smads (Smads inhibitors): Smad 6 and 7. They are able to interact with membrane receptors by forming stable complex with activated T*β*RI, and thus impairing their interaction with the R-Smad (inhibition of their phosphorylation). Smad7 expression is induced by TGF-*β*, leading to inhibition of the cellular response to this cytokine [26]. Smad7 has been shown to promote recruitment of E3 ubiquitin ligases (including Smad ubiquitin regulatory factors (SMURF1, SMURF2, PRAJA, WWP1, and Nedd4-2) into the receptor complex [8]. Binding of Smad7 and SMURF to the receptor complex also results in competitive inhibition of Smad2/3 binding to T*β*RI [25]. TGF-*β*1 signaling is also attenuated by other proteins, which interact with Smad7, like STRAP or YAP65 [5]. Therefore, there is an autoregulation of the negative feedback mechanism. Anti-TGF-*β* activity of Smad7 can be negated by AMSH2 or Arkadia [28]. Under disease conditions, Smads also interact with other signaling pathways, such as the mitogen-activated protein kinase (MAPK) and nuclear factor-*κ*B (NF-*κ*B) pathways [29, 30].

It is well known that TGF-*β*1 also signals in a Smad-independent manner (noncanonical pathways), by induction of other pathways, such as the extracellular signal-regulated kinase 1/2 (ERK1/2) and the p38 MAP kinase (p38 MAPK) [5, 18]. MAPK pathways are showing direct function in signal transduction of TGF-*β*1-modulated cellular migration and invasion [1]. At present, the Smad-independent pathways are known to include ShcA, RhoA-Rock1, RAC/CDC42, RAS, TRAF6-TAK1-p38/JNK, PI3 K, PAR6, MAP3 K1, DAXX, and PP2A [5, 6, 8, 18, 28]. RhoA-Rock1 signaling with engagement of TGF-*β*1 is required for the EMT [9, 31].

## 4. TGF-***β***1 Paradox in Cancer

The role of TGF*β*-1 in cancer progression has been shown to be multifaceted, depending on the tumor stage. This cytokine acts as a potent growth inhibitor. It has been shown to inhibit epithelial cell cycle progression and promote apoptosis that together significantly contribute to the tumor suppressive role during carcinoma initiation and progression [5, 25, 32, 33]. However, the ability of TGF-*β*1 to induce and promote EMT associates this cytokine with increased tumor cell motility and invasion [34]. Thus, TGF-*β*1 is also regarded as a metastasis inducer, participating in malignant progression and angiogenesis [1, 5, 14, 25, 27, 33, 35–37]. These contrasting, dichotomous TGF-*β*1 behaviors in cancer development and progression are known as “TGF-*β*1 paradox” [17, 18].

Currently the link between tumor progression and modification of tumor microenvironment (interaction between carcinoma cells and adjacent cell populations) is under profound investigation. The role of TGF-*β*1 in this context has been studied by many research groups [5]. One of the predominant stromal-epithelial axes, associated with the regulation of cancer progression, involves carcinoma-immune cell interactions within the tumor microenvironment [38]. Specifically, TGF-*β*1 has been shown to suppress the antitumor activity of T cells, NK cells, neutrophils, monocytes, and macrophages that are known to have a significant role in the regulation of tumor progression [39].

Both tumor suppressor and tumor-promoting activities of TGF-*β*1 have been clearly demonstrated in a variety of genetically modified mouse lines, in which the TGF-*β*1 signaling pathway is ablated or modified [36]. These studies support a model, in which TGF-*β*1 inhibits the development of early, benign lesions, but promotes invasion and metastasis, when the tumor suppressor activity is overridden by oncogenic mutations in other pathways [5, 10, 36]. Changed expression of growth factors and their receptors has been shown to play an important role in the neoplastic formation and tumor progression.

## 5. TGF-***β***1 Role in Early Stages of Tumor Progression

In normal physiological conditions, TGF-*β*1 is a potent inhibitor of growth of many cell types (cytostatic effect as well as the effect on apoptosis), including neoplastic cells [26]. The TGF-*β*1 cytostatic responses primarily target G1 events, by regulating the expression of several genes promoting cell cycle arrest. In normal epithelial cells, TGF-*β*1 induces expression of p15Ink4b, which inhibits Cyclin D-Cdk 4/6 complexes, and of p21, which inhibits Cyclin E/A-Cdk2 complexes. In response to TGF-*β*1, activated Smad-FoxO (FoxO1, FoxO3, and FoxO4) transcriptional complexes target a region of the p21 promoter and mediate the induction of p21. On the other hand, TGF-*β*1 induction of p15Ink4b by activated Smad-FoxO complexes additionally requires C/EBP *β* for their response to TGF-*β*1 [26]. The TGF-*β*1 cytostatic effect also involves transcriptional repression of the growth-promoting transcriptional factors c-Myc and inhibitors of differentiation (ID 1,2,3) [8, 9]. TGF-*β*1 downregulates the expression of c-Myc in MECs and human skin keratinocytes. Repression of c-Myc is mediated by binding of a transcriptional repression complex containing Smad, E2F4/5, p107, and C/EBP*β* to the TGF-*β*1 inhibitory element in the proximal region [26].

In the early stages of cancer development, cancer cells respond to antimitotic effect of TGF-*β*1. TGF-*β* controls cell proliferation mainly by inhibiting cell cycle progression through G1-arrest, by inducing or activating cdk inhibitors such as p16INK4A, p15INK4B, p21CIP1, and/or p27Kip1 [6, 8]. When the tumor cells are entering the phase of uncontrollable growth, the majority of them lose sensitivity to this inhibitory effect [40]. Surprisingly, this occurs despite the presence of the TGF-*β*1 receptors on the tumor's cell membranes. Furthermore, these cancer cells also begin to secrete TGF-*β*1. On the other hand, it has been noted that in early development increased TGF-*β*1 expression leads to inhibition of mammary epithelial outgrowth* in vivo* [41]. Also a decreased incidence of tumorigenesis induced by infection with the mouse mammary tumor virus in mammary epithelium with TGF-*β*1 expression was reported [41].

Indirect regulation of tumor suppression by TGF-*β*1 can be correlated with blockage of paracrine factor production (stromal cell-derived factor-1, SDF-1) in the tumor stroma. While the impact of the stromal fibroblast on tumor progression has been known since early studies on breast cancer, the role of TGF-*β* in this process emerged from mouse models in which TGF-*β* signaling was impaired in stromal fibroblasts [9].

The TGF-*β*1-dependent immunosuppressive activity stimulates angiogenesis, increasing the affinity of cancer cells to cell adhesion molecules, and creates a microenvironment favorable to tumor growth and its metastasis—increasing cancer cells invasiveness. Additionally, TGF-*β*1 induces death of the surrounding healthy cells and thus eliminates their effect designed to inhibit tumor growth. It appears that cancer cells need higher TGF-*β*1 concentrations than normal cells to receive TGF-*β*1-anti-mitotic stimulus [12]. The results of clinical and experimental studies indicate that the molecular background of the lack of cell response to TGF-*β*1 during malignant transformation is the mutations in the T*β*RII receptor and/or within the Smad proteins [26, 40].

## 6. TGF-***β***1, T***β***R and Smad Mutations and Inactivation

Many investigations among different human populations have detected different mutations in the TGF-*β*1 [13]. Amani et al. [13] reported, for the first time, that the TGF-*β*1 haplotype “GTGCCGC” might be associated with BC in Iranian woman. Several somatic mutations that disrupt the TGF *β*1-Smad signaling pathway have been reported in human breast tumors [26, 42, 43]. These mutations may affect the different aspects of BC, including its occurrence, prognosis, progression, and metastasis. Single nucleotide polymorphisms (SNP) of genes are widely examined. 29T→C coding cSNP (Leu10Pro, rs1800470) is mostly studied in BC field, and it is the most prevalent polymorphism of TGF-*β*1. The Pro-allele is considered a “high-activity” (hypermorphic) allele compared to the Leu-allele [44]. A large study of the Breast Cancer Association Consortium (BCAC) has reported an association of the Pro-allele with a moderate, but significantly increased, BC risk (e.g., Pro/Pro versus Leu/Leu: OR, 1.16; 95% confidence interval, 1.08–1.25). Other studies have either reported an increased risk, an unaltered risk, or even a decreased risk associated with the Pro-allele [44]. The findings by Taubenschuß et al. [44] indicated that the Pro-allele may also lead to higher TGF-*β*1 secretion* in vivo* but that the observed effects on serum levels were less pronounced and more heterogeneous than* in vitro*. In this work [44] the L10P SNP of* TGF*β*1* was genotyped in 274 breast cancer patients and 252 female controls. The frequency of the Pro-allele was 40.0% in patients and 42.3% in controls. The Pro/Pro genotype was slightly less frequent in BC patients than in controls (16.1% and 19.0%, resp.). The fraction of patients with the Pro/Pro genotype tended to be increased in several patient subgroups associated with advanced cancer progression and/or poor prognosis. The same conclusions can be found in other studies [45–47]. That could be the evidence on the dual role of TGF-*β*1 in different cancer stages and cancer subclasses. It has been suggested that the Pro-allele is associated with a reduced risk of* in situ* tumors, but an increased risk of invasive BC; or with a reduced risk of early-stage invasive BC, but an increased risk of BC with advanced stages [48]. The BCAC study has reported higher odds ratios associated with the Pro-allele in patients with high tumor grade and stage, and negative ER and PR status, although only the latter association was statistically significant. Similarly, the Pro-allele was associated with a reduced risk of early-stage BC, but an increased risk of BC with advanced stages [44].

The majority of tumor-derived mutations in Smad2 and Smad4 cluster are in the MH2 domain. Some of them have been shown to disrupt TGF-*β*1 signaling by blocking receptor dependent phosphorylation, or by disrupting oligomerization of the Smads. Smad4 harboring the missense mutation in the MH1 domain exhibits accelerated induction of ubiquitin dependent proteasomal degradation in comparison with wild-type Smad4. Skp2 (S-phase kinase-associated protein2) is upregulated in various human cancers and promotes the ubiquitination-dependent degradation of these Smad4 cancer mutants [26]. In the case of Smad3 no mutation has been found in human cancer. STAT pathway and NF-*κ*B pathway also induce Smad7, thus, tumor cells with high activity of those pathways might evade the TGF-*β* cytostatic responses through overexpression of Smad7. The important negative regulators of TGF-*β*1 signaling are SKI and SnoN, which interact with Smad-2 -3 and -4. Increased expression of SKI or SnoN has been implicated in the progression of ER-positive (ER+) breast carcinoma. When SKI and SnoN are downregulated (RNAi) TGF-*β*1-mediated growth inhibition in BC is restored [26].

At present, a substantial number of correlative data demonstrate that TGF-*β*1 signaling components, including T*β*RI, T*β*RII, Smad2, and Smad4 are often lost in human cancer. Consistent with its tumor suppressor role, many cancers lose or attenuate TGF-**β**-mediated antimitogenic action by mutational inactivation of TGF-**β** receptors or Smads [49]. Studies using transgenic mice with conditional knockout of T**β**RII indicate that loss of T**β**RII in the context of polyomavirus middle T antigen (PyVmT) expression results in a shortened median tumor [49]. Mutations and loss of type I and type II TGF-*β*1 receptor expression have been detected in most types of common cancer, including those that occur in the biliary tract, bladder, breast, colon, esophagus, stomach, brain, liver, lung, ovary, pancreas, and prostate [5, 10, 27, 50]. In human BC, the alterations of TGF-*β*1 signaling molecules are relatively rare, except for T*β*RII downregulation [6, 51]. Pathological studies of archived breast samples, including benign lesions, ductal carcinoma* in situ* (DCIS), and invasive mammary carcinomas (IMC), indicated that T*β*RII downregulation is correlated with progression and aggression of both* in situ* and invasive breast carcinoma [52]. Recent studies have shown that silencing of the* T*β*RII* gene can occur through methylation in human breast carcinoma cells [53]. In human MECs and human mammary carcinoma cell lines the expression of TGF-*β*1, T*β*RI, and T*β*RII was concurrently suppressed by methylation, and these genes could be coordinately reinduced upon demethylation [54]. T*β*RII inactivation enhances the invasiveness of premalignant or low-grade breast tumor cells but reduces the metastasis of high-grade tumors [55]. In the opposite, T*β*RIII may act as a suppressor of BC, as the decrease or loss of T*β*RIII expression occurs in approximately 90% of BC at mRNA levels and 70% at protein levels. Additionally, T*β*RIII loss occurs at substantially high levels in advanced, invasive breast carcinomas. Therefore, its loss may be a negative prognostic factor for patients with invasive BC [56].

## 7. Role of TGF-***β***1 in Late Stages of Tumor Progression and Metastases

In the late stages of tumor progression TGF-*β*1 is changing its action into tumor promoter. Several studies have shown a broad range of potential TGF-*β*1 effects on cancer metastasis [21]. Immunostaining analyses revealed a correlation between TGF-*β*1 expression and metastasis in breast, colon, and prostate cancer. In addition, the intensity of TGF-*β*1 staining in invading lymph node metastases was higher in breast and colon cancers than in the primary tumors [9]. Metastasis is a multistep cascade process, including EMT, cell migration, invasion, intravasation, and extravasation from the circulation [23, 26, 52, 57]. Cheng et al. [58] demonstrated using an* in vitro* assay that cancer cells cultured under fibroblast-conditioned medium showed increased proliferation and motility, indicating the role of stromal TGF-*β*1 signaling in neoplastic progression. Conversely, attenuation of carcinoma cell response to TGF-*β*1 by a dominant-negative type II receptor transgene (dnT*β*RII) significantly reduced tumor latency in the presence of TGF-*α* expression in mammary epithelium [52]. This indicates that specific TGF-*β*1 signaling in carcinoma cell is able to promote tumor cell invasion. It has been suggested that Smads are involved in the antitumor process, while the Smad-independent pathways have been implicated in induction of tumor progression [27, 59]. However, recent data also demonstrate that Smad-dependent pathways are involved in the tumor-promoting activities of TGF-*β*1. Smad-3 and -4 are necessary for the metastatic expansion of bone neoplasms, whereas Smad-2 in the case of lung, liver, and brain tumors, respectively [9, 60]. Approximately 40% of the human breast cancers show a positive TGF-*β* gene response signature, that is context dependent and appears more in ER-tumors (as opposed to ER+ tumors) and in lung metastasis (as opposed to bone metastasis) [8, 60]. The mechanism of the TGF-*β* induced lung metastasis in breast cancer is related to the induction of the angiopoietin-like 4 (*ANGPTL4*) gene by TGF-*β* Smad-dependent signaling in the primary tumor, enabling the cells which leave the breast to disrupt the lung capillary walls. The fenestrated capillaries of the bone marrow do not have any advantage from the action of* ANGPTL4*, and that might explain why the impact of TGF-*β* is directed to lung and not to bone metastasis [6, 8, 9, 27, 60].

At least seven genes (*IL-11*,* CTGF*,* CXCR4*,* MMP-1*,* PTHrP*,* VEGF,* and* PTGS2/COX2*) have been identified as drivers of human breast cancer bone metastases in the MDA-MB-231 model, and each of these genes is transcriptionally regulated by and dependent on TGF*β* signaling* in vivo* [20]. TGF-*β* is a major contributor to the bone metastases, and TGF-*β* is released from bone matrix by the activated osteoclasts that degrade the bone matrix. Secreted TGF-*β* stimulates releasing of other osteolytic cytokines, such as parathyroid hormone related protein (PTH-rP), IL-11, and CTGF (Connective Tissue Growth Factor) from the metastatic cells to maintain the metastatic process.

The work of Micalizzi et al. [61] indicated that Six1 may be a critical mediator of the switch in TGF-*β*1 signaling from tumor suppressive to tumor promotional. However, the mechanism by which Six1 impinges on the TGF-*β*1 pathway remains unclear. Scientists [61] have shown in* in vivo* experiment that a target for Six1 is T*β*RI, and Six1 overexpression is required to switch TGF-*β*1 signaling to the prometastatic phenotype, showing that induction of EMT is not sufficient to induce experimental metastasis. Instead, T*β*RI upregulation in the absence of Six1 overexpression actually inhibited metastatic spread* in vivo* in an experimental metastasis model. Thus, Six1 is regarded as a determinant of TGF-*β*1 function in BC. Six1 is misexpressed in numerous cancers including breast cancer. In human BC, Six1 correlates with advanced disease and adverse patient outcomes [62].

Several studies on mouse models showed that MECs specific expression of activated TGF-*β*1 ligand or expression of active T*β*RI could enhance BC-associated lung metastases* in vivo *[57]. In the study of Darakhshan and Ghanbari [35] administration of tranilast with tamoxifen (TAM) downregulated the expression of TGF-*β*1, *β*-2, and *β*-3, as well as T*β*RI and T*β*RII in breast cancer cells (MCF-7 and MDA-MB-231 human breast cancer cell lines). T*β*RIII is a suppressor of BC progression and when its expression is restored, invasion, angiogenesis, and metastasis are inhibited* in vivo* [56]. In the study of Darakhshan and Ghanbari [35] tranilast and TAM slightly increased the expression of T*β*RIII.

## 8. TGF-***β***1 and EMT

BC starts as a local disease and can metastasize to distant organs. The conversion of early stage tumors into invasive malignancies has been associated with the activation of EMT, defined as changes in cell phenotype from an epithelial to a mesenchymal state, which is both a fundamental event and a hallmark in tumorigenesis [26]. Changes during EMT lead to the transition from a polarized epithelial phenotype to an elongated fibroblastoid phenotype, then cells degrade the ECM, and show invasive behavior [63–65].

TGF-*β* function is often accompanied by desmoplastic and fibrotic reactions, which elicit dramatic changes in the biomechanical properties of the tumor microenvironment. The elastic modulus of stroma housed within breast carcinomas is approximately 10 times more mechanically rigid than that of adjacent normal breast tissues. TGF-*β* potentiates these biomechanical reactions by stimulating the expression and secretion of a variety of ECM components, such as collagen I and fibronectin from stromal fibroblasts, and of ECM cross-linking enzymes, such as lysyl oxidase from mammary carcinoma cells. The formation of these rigid mammary tumor microenvironments promotes metastatic progression in breast cancers and also predicts poor clinical outcomes in patients harboring metastatic disease [66].

TGF-*β*1 was shown to play important regulatory role in EMT [8, 16, 67]. Identification of TGF-*β* as a major inducer of EMT came from* in vitro* studies on cell cultures. Treatment of normal mouse breast epithelial cells with TGF-*β* changes the cuboidal shape to an elongated spindle, accompanied by a decrease in epithelial markers and increased expression of mesenchymal markers [9, 28]. It induces the increase in cell size and protein content during EMT (as a result of mTOR activation) [68–70]. Its signaling downregulates claudins, occludins, and ZO1, followed by tight junctions degradation [63]. TGF-*β*1 also upregulates integrin-linked kinase (ILK), increasing cellular motility [23, 63, 69, 71]. Expression of integrins (e.g., *α*5, *α*v, *β*1, *β*3, *β*5) that bind the ECM is also enhanced by TGF-*β* cell signals. Ligation of these integrins (*α*2*β*1 : collagen, *α*5*β*1 : fibronectin, *α*v*β*3 or *α*v*β*5 : periostin) induces the production of TGF-*β*, leading to a feed forward loop between tumor cells and the ECM [72].

TGF-*β*1-induced EMT is largely studied using NMuMG murine mammary epithelial cells, because these MECs are known to undergo EMT readily apparent 36 h after TGF-*β*1 treatment [68, 69, 73]. In this kind of EMT canonical Smad signaling, as well as Smad-independent signaling, (through small GTPases and the ERK1/2 and p38 MAPK pathways) is integrated [25, 28, 73]. Increased expression of Smad3 and Smad4 in the presence of constitutively active T*β*RI enhances induction of EMT [9]. Smads act as transcription factors of EMT regulators, such as Snail/Slug/Twist, Cripto-1, FOXC2, and Six1 [6, 28, 61, 74]. For example, activation of Smad2/3 by TGF-*β* in MECs induces the expression of the nuclear high mobility group A2 (HMGA2), which promotes EMT by stimulating the expression of Snail1, Snail2/Slug, and Twist, and by inhibiting the expression of ID2 [28]. The expression of E-cadherin is repressed by TGF-*β*1-mediated SNAIL-Smad3/4 complex which negatively regulates E-cadherin in breast epithelial cells [24, 28, 75]. E-cadherin is also repressed by HMGA2, TBX3 (The T-box transcription factor) [76] and ZEB 1/2 (Zinc finger E-box-binding homeobox 1/2) [18, 25]. Furthermore E-cadherin is lost during EMT and cancer progression [63, 65].

Matrix rigidity converts TGF-*β* from a proapoptotic molecule to an inducer of EMT in NMuMG and MDCK cells, by enhanced coupling of TGF-*β* to the PI3 K/Akt pathway [74]. Lamouille and Derynck [68] have used the NMuMG cell model and observed that the typical loss of epithelial phenotype with concomitant acquisition of the spindle-shaped fibroblastoid phenotype, induced in the presence of TGF-*β*, was accompanied by an increase in cell size and protein content and correlated with rapid mTOR activation. The authors observed also that rapamycin inhibited the migration and invasion of cells after TGF-*β*-induced EMT, which is in agreement with recent observations that rapamycin inhibits the induced motility of some cancer cells [28, 77]. As PI3 K and mTOR activities are commonly upregulated in various cancers, PI3 K inhibitors and rapamycin analogues are investigated as inhibitors of cancer progression in preclinical and clinical trials [68, 69].

Studies suggest that Twist, Snail, and TGF-*β* may induce the expression of cell surface markers associated with cancer stem cells and these cells share high homology to bone marrow-derived mesenchymal stem cells [28].

## 9. TGF-***β***-Induced EMT and microRNA Regulation

There is a double negative feedback loop between microRNA: miR-200 (transcriptional targets of ZEB), miR-205, and ZEB, which allows for the plasticity existing between the cell's epithelial and mesenchymal state [28, 74, 78–80]. In addition, the same microRNAs are frequently downregulated in invasive human breast cancer cells that exhibit a mesenchymal-like morphology [28]. Recent studies demonstrated that highly metastatic 4T1 breast cancer cells are more epithelial-like as compared to their isogenic and nonmetastatic 4T07 counterparts [28]. Amongst the many unique differences between these two isogeneic cell types is the reexpression of miR-200 in metastatic 4T1 cells, leading to the synthesis and secretion of metastasis-promoting proteins necessary for metastatic outgrowth [28, 80]. In contrast to the miR-200 family, metastatic breast cancers were found to preferentially upregulate the expression of miR-10b, which promotes the invasion and metastasis of malignant MECs both* in vitro* and* in vivo* [28, 81].

In the study of Xu et al. [78] TGF-*β*1 secretion resulted in an increased level of ZEB1 transcription in MCF7 cells, that could reach a point, where ZEB1 transcription and protein accumulation could overcome the repression caused by miR-200, resulting in the progression of the EMT. The downregulation of paracrine TGF-*β*1 signaling could reduce ZEB1 and ZEB2 expression, upregulate miR-200b and miR-200c, and finally inhibit the progression of the EMT. In breast cancer progression, miR-221/222 expression is increased in aggressive basal-like subtype breast cancers. In these tumors, miR-221/222 directly represses the expression of the GATA family transcription factor TRPS1, a repressor of ZEB2. By this action miR-221/222 is promoting downregulation of E-cadherin expression, and EMT-associated increased cell migration and invasion [82].

Conversely, miR-520/373 members act as tumor-suppressive miRNAs, and increased miR-520c or miR-373 expression inhibits the invasive behavior of breast cancer cells* in vitro* and* in vivo*, in part by targeting T*β*RII [83]. Direct repression of Smad7 can be seen in action of miR-106b-25 cluster [84]. In the work of Kong et al. [85] administration of TGF-*β* to normal murine MECs (NMuMG) induced miR-155 expression through a Smad4-dependent mechanism. Once expressed, miR-155 abrogated MEC expression of RhoA and prevented their ability to undergo EMT in response to TGF-*β* [28, 74, 85]. miR-181a expression is also highly associated with the development of metastatic disease in BC. In the study of Taylor et al. [66], TGF-*β* treatment of NMuMG cells induced EMT resulting in a significant upregulation of miR-181a expression. Furthermore, it was demonstrated that inactivation of miR-181a prevented the loss of E-cadherin expression stimulated by TGF-*β*.

## 10. TGF-***β***1 and ECM Degradation

ECM degradation is an important part of the metastatic process. In breast cancer destabilization of p53 by Mdm2 (E3 ubiquitin-protein ligase Mdm2) is a pivotal step in EMT. Late-stage metastatic breast cancer progression can be correlated with TGF-*β*1-induced expression of Mdm2. Furthermore, in bones (common site of BC metastasis) cancer cells are able to activate osteoclasts influencing the extracellular matrix degradation, and releasing growth factors stored there (TGF-*β*, IGF, BMP). TGF-*β*1 was shown to stimulate cancer cells for osteolytic cytokines induction (e.g., PTHrP—stimulator of NF*κ*B ligand RANKL production), which enhanced the osteoclast differentiation [9, 26]. In the lung metastasis by breast cancer cells, TGF-*β*1 signaling in the tumor microenvironment primed cancer cells for pulmonary metastasis [26, 86].

Essential regulators of ECM degradation are matrix metalloproteinases (MMPs) [63, 65], their specific inhibitors TIMPs (tissue inhibitors of MMPs), and the membrane-associated MMP inhibitor (RECK). The balance between these molecules regulates motile and invasive capacities [1, 87]. MMP-2, MMP-9, MMP-14, and TIMP-2 are linked with BC progression [88, 89]. Many scientific reports have suggested a crucial function of TGF-*β*1 as a modulator of MMPs [90, 91]. TGF-*β* enhances the tumorigenicity and invasiveness of breast cancer cells by inducing their expression of MMPs 2 and 9 [28]. Gomes et al. [1] demonstrated for the first time that TGF-*β*1 is able to modulate MMP, TIMP, and RECK expression in MDA-MB-231 human breast cancer cells through ERK1/2 and p38MAPK pathways. Both of these transducer pathways were essential for the TGF-*β*1-enhanced migration and invasion phenotypes; however, each mediated the TGF-*β*1 signal for MMPs and their inhibitors in a specific manner. This study demonstrated that, similarly to MMPs, TIMPs, and RECK, the expression of T*β*RI and T*β*RII was higher in the most aggressive cell line (MDA-MB-231), as compared to the less invasive ones, except for T*β*RI, that was also highly expressed in ZR-75-1 cells [1]. Kim and collaborators [92] suggested that TGF-*β*1 also induces invasion in premalignant breast cancer cells (MCF10A), by upregulation of MMP-2 and MMP-9 [92]. Subsequent reports also indicated that MMP-2 and MMP-9 are essential in the TGF-*β*1-increased invasion of MCF10 cell series in a 3D* in vitro* model [24, 93].

The results suggest that TGF-*β*1 could suppress primary tumor growth while promoting metastasis through EMT of the responding carcinoma cells. In a mouse model of mammary carcinoma, with complete ablation of TGF-*β*1 response in mammary epithelium, a decrease in tumor latency was observed. Furthermore, a striking increase in pulmonary metastasis was also clearly demonstrated [94]. In this model the loss of TGF-*β* signaling in the mammary carcinoma cells caused also an increased abundance of smooth muscle actin positive stroma, tumor cell heterogeneity, and tumor cell survival. Additionally, TGF-*β* regulated chemokine expression resulting in carcinoma-immune cells, which could be related to mammary carcinoma cell metastasis [94, 95]. A reduced response of tumor cells to TGF-*β* signaling is often accompanied by an increase in secretion of this ligand. In breast cancer patients with poor prognosis [94], TGF-*β*1 levels were often elevated in plasma, tumor cells, and associated stroma [23, 96].

## 11. Mesenchymal-Epithelial Transition (MET)

Following EMT, metastatic cells can revert back and reacquire epithelial properties, similar to cells in the primary tumor [97]. This process is called mesenchymal-epithelial transition (MET) and contributes also to formation of tissues and organs during development [82]. It is less characterized than EMT, but MET can correlate with the establishment of secondary tumors following metastasis. Interestingly, members of the miR-106b-25 cluster can promote a MET-like process and enhance the induction of iPSCs (Induced Pluripotent Stem Cells) reprogramming through targeting T*β*RII [82], whereas, miR-200 family has been implicated in promoting MET through their ability to repress the expression of ZEB1 and ZEB2, leading to upregulated E-cadherin expression [74].

## 12. TGF-***β***1 and Immune Cells in Tumor Microenvironment

TGF-*β*1 mediates recruitment of tumor promoting myeloid cell populations. Mammary carcinoma cells specific ablation of TGF-*β*1 signaling led to enhanced metastasis and was associated with an increased myeloid cell infiltrate in mice [94]. Also GR1+ CD11b+ and F4/80+ myeloid cells were recruited to the leading edge of tumors exhibiting a carcinoma cell specific ablation of TGF-*β*1 responsiveness [98]. It was correlated with increased expression of Cxcl1 and Cxcl5 in TGF-*β*1 signaling deficient tissues* in vitro* and* in vivo. In vivo *studies showed that CxcR2 signaling significantly contributed to enhanced metastasisobserved from the TGF-*β*1 signaling deficient mammary carcinoma cell population, when compared with the control mammary carcinoma cells [98]. When TGF-*β*1 is available in the tumor microenvironment for stimulation of adjacent cell populations, including immune cell infiltrates, it can have a significant impact upon antitumor activity of T cells [5]. IL-2 dependent T-cell signaling has been reported to involve suppression of IL-2 production by Smad3. TGF-*β*1 was also shown to regulate T-cell growth arrest (p21Cip1 and p27Kip1 are known TGF-*β*1 targets) in the presence of exogenous IL-2 and IL-4, that would normally promote proliferation. TGF-*β*1 is also known to suppress T-cell mediated tumor rejection [3]. TGF-*β*1 can cause host macrophages to become suppressors of CD4+ T-cell proliferation. It has recently been shown that the CD4+ CD25+ regulatory T-cell population can provide a significant source of TGF-*β*1, that is responsible for attenuation of tumor antigen expanded CD8+ cytotoxic T cells (CTLs) [27, 99]. At present a number of studies have clearly demonstrated that TGF-*β*1 can suppress cytotoxic T-cell differentiation and cytotoxic T-cell mediated lysis of carcinoma cells [100]. Additionally, TGF-*β*1 was shown to prevent the expression of granzyme A, granzyme B, perforin, Fas ligand (FasL), and interferon-gamma-promoters of CTLs cytotoxicity [8, 100, 101]. Granzyme B and interferon-gamma expression was directly linked to Smad transcription factors [101]. Moreover, it has been reported that TGF-*β*1 stimulation inhibits NK cell and neutrophil effector functions, which contributes to tumor progression in a permissive microenvironment. In addition, TGF-*β*1 has been shown to suppress MHC I and MHC II expression in a number of cell populations. Importantly, the TGF-*β*1 dependent decrease of MHC I expression in tumor cells results in reduced tumor cell lysis by NK cells, thereby enhancing tumor growth and metastasis [102]. TGF-*β*1 is one of the most potent known chemoattractants for human peripheral blood neutrophils that also inhibits their ability to suppress tumorigenesis and potently regulates the interaction between neutrophils and other cell populations within the tumor microenvironment [5]. Neutrophils function in the tumor microenvironment is the recognition and destruction of carcinoma cells expressing FasL. In the presence of TGF-*β*1 neutrophils exhibit a decreased ability to eliminate such cells [5]. TGF-*β*1 is also promoting recruitment of monocytes, and it has been suggested to promote monocyte to macrophage differentiation [5]. TGF-*β*1 is able to block both the priming by interferon-*γ* and triggering by lipopolysaccharide (LPS) of macrophages, necessary for the efficient killing of tumor cells by macrophages [5]. Conversely, tumor necrosis factor alpha (TNF-*α*) cytotoxicity is functioning independently of TGF-*β*1 influence. TGF-*β*1 pretreatment of carcinoma cells attenuates both the cytotoxicity and cytostatic ability of macrophages* in vitro* [5]. TGF-*β*1 stimulation of macrophages has been shown to attenuate macrophage-associated suppression of CD4+ T-cell proliferation [5]. TGF-*β*1 also suppresses MIP-1a, MIP-2, CXCL1, IL-1*β*, IL-8, GM-CSF, and IL-10 expression. In monocytes, TGF-*β*1 has been shown to promote expression of IL-1 and IL-6 and suppress oxygen free radical production [5]. It plays also a pivotal role in inducing the differentiation of Tregs (CD4+ CD25+ Foxp3+ regulatory T cells) [103], which are thought to be the main obstacle tempering antitumor immunity and immunotherapy. Their localization and the infiltrating patterns vary in BC and have different impacts on tumor progression but can be prognostic factor for BC [8].

## 13. Anticancer Therapeutic Strategies against TGF-***β***

Due to its growth-suppressive effects, in the past, TGF-*β* has been regarded as an attractive cytokine for the treatment of cancer. Therefore, studies were initiated to explore the potential role of TGF-*β* as an adjuvant for chemotherapy. TGF-*β* was able to protect normal cells and sensitize tumor cells towards standard chemotherapy in some preclinical models [9]. Given TGF*β*'s pleiotropic effects on both tumor cells and host cells, and its presumed role in tumor metastasis, detailed assessment of antitumor effects of TGF*β* antagonists can only be accomplished by using models of metastatic mammary cancer: the murine metastatic mammary cancer cell lines 4T1 (Balb/C), EMT6 (Balb/C) and R3T (129S1), and the human metastatic MDAMB-231, MDA-MB-435, MCF10ACA1A, and MX-1 cell lines that are inoculated into immunodeficient mice [20].

With current knowledge about the involvement of TGF-*β* in progression and metastasis of cancer, there are 3 different approaches against TGF-*β*, which have therapeutic potential.

(1) Using antisense molecules to prevent TGF-*β* synthesis on ligand level; (2) blocking the ligand-receptor interactions by using ligand traps (monoclonal antibodies and soluble receptors) and antireceptor monoclonal antibodies; (3) inhibiting signaling cascade on the intracellular level (with the use of TGF-*β* receptor kinases inhibitors and peptide aptamers) [6–9]. For each of these approaches, several drugs have been developed and are either in nonclinical or in early stages of clinical investigation. A few examples can be found for BC therapy.

Antisense molecules are single stranded oligonucleotides (13–25 nucleotides) [8]. Since TGF-*β* production is usually increased during tumor progression, blocking its synthesis and TGF-*β*-mediated gene expression have the potential to reduce excess TGF-*β* levels within the tumor microenvironment. Antisense mediated inhibition of TGF-*β*1 gene expression has been shown to be effective in reducing malignant properties of mouse fibrosarcoma cells and murine 4T1 cells [104]. Some of the tested molecules were shown to be efficient in treatment of pancreatic cancer (AP12009 Trabedersen) or prostate carcinoma (AP11014 and AP15012) [8, 9, 104].

Ligand traps can control excess of TGF-*β* production in tumor microenvironment. A neutralizing monoclonal antibody (mAB), 1D11 (Genzyme Corp.,Sanofi), that binds TGF-*β* 1, 2, and 3, resulted in suppression of lung metastasis in metastatic breast cancer mouse model, mainly by increasing the antitumor response of CD8+ T cells [105]. It also gave decreased bone loss by reduced expression of PTHrP and its regulator Gli2 [50]. In the* in vitro* experiments of Tan et al. [20], which assessed the efficacy of the murine anti-TGF*β* monoclonal antibody 1D11, the experimental metastasis models were used: bone-tropic and lung-tropic MDA-MB-231 human breast cancer cells sublines (preferentially metastasize to lungs: MDA-231-4175TR or bones: MDA-231-SCP2TR and 2860TR). Treatment with 1D11 was able to block TGF*β*-induced phosphorylation of the receptor-associated Smads, Smad-2 and -3, in each of these cell lines. While 1D11 had no effect on cell growth* in vitro*, it inhibited TGF*β*-stimulated tumor cell migration and invasiveness into Matrigel. Treatment with 1D11 antibody significantly reduced the burden of MDA-231-SCP2TR or 2860TR-derived metastases to bones, as well as MDA-231-4175TR-derived metastases to lungs by ~40%.

In preclinical trials another mAB, 2G7, showed efficacy in inhibiting breast cancer metastasis by increasing NK cells activity and preventing radiation induced acceleration of metastases [20, 104, 106–108]. Genzyme had developed three fully humanized mABs: GC-1008 (Fresolimumab), CAT-152 (Lerdelimimab), and CAT-192 (Metelimumab), which were tested in clinical trials [3, 104]. GC-1008 is capable of neutralizing all three TGF-*β* isoforms. CAT-152 is in phase III clinical trials for some metastatic tumors [3, 59]. GC-1008 was tested in phase I/II clinical trials. Two trials of GC-1008 are in recruitment phase: Fresolimumab and radiotherapy in metastatic breast cancer (NCT01401062), and safety and imaging study of GC1008 in glioma (NCT01472731). The other two mABs have not been tested yet on cancer patients.

Another way of blocking TGF-*β* is to use soluble receptors. For example soluble T*β*RII and T*β*RIII have been tested in preclinical studies in breast and pancreatic cancer metastasis [104, 109–111]. Muraoka et al. [109] have shown that systemic administration of Fc:T*β*RII increased apoptosis of primary mammary tumors expressing PyMT (polyoma middle T-antigen) and reduced tumor cell motility, intravasation, and lung metastases. Similarly, Fc:T*β*RII also inhibited metastases from transplanted 4T1 and EMT-6 mammary tumors in syngeneic BALB/c mice. Expression of soluble T*β*RII reduced BC and pancreatic cancer metastasis. No clinical trials have been undertaken with these soluble receptors until now.

In signal transduction blockade two different strategies can be proposed: the use of receptor kinase inhibitors, and targeting the intracellular TGF-*β* signaling pathway molecules, such as Smads, with peptide aptamers [6, 8, 50]. Peptide aptamers are small molecules containing a target binding site and a scaffolding domain that impedes the function of the target [8].

Targeting receptor kinases has been intensively investigated, because such drugs are easy to produce and can be administrated orally [50]. Ki26894, SD-208, and LY364937 are T*β*RI inhibitors, which appeared to be promising in terms of inhibiting metastasis to bone. Experiments were conducted using breast and gastric cell lines* in vitro* [112, 113] and xenografts mouse model* in vivo* [112, 114]. In the experiments of Ehata et al. [112] treatment with Ki26894 blocked TGF*β* signaling in MDA-MB-231-D cells, which was detected by suppression of Smad2 phosphorylation and inhibition of TGF*β*-responsive target genes activity. Moreover, Ki26894 decreased the motility and the invasion of MDA-MB-231-D cells induced by TGF*β in vitro*. Systemic Ki26894 treatment initiated 1 day before the inoculation of MDA-MB-231-D cells into the left ventricle of BALB/c nu/nu female mice resulted in decreased bone metastases and prolonged survival compared to vehicle-treated mice.

SD-208 has been shown to inhibit growth of primary tumors and pulmonary metastasis in tests with two murine mammary carcinoma lines, R3T and 4T1 [20, 104]. SB-431542, the most widely used T*β*R1 inhibitor, has been shown to inhibit tumor metastasis in breast cancer [3, 115], glioma, and renal cell carcinoma in the preclinical stage [3]. LY2109761 is a small molecule inhibiting the kinase activity of both T*β*RI and T*β*RII. This compound inhibits metastasis formation in mouse models of breast cancer [8, 9, 104, 116]. LY2157299 (Eli-Lilly & Co) is a T*β*RI kinase inhibitor that reduces growth of lung and breast cancer cell lines and has been shown to inhibit primary tumor growth induced by the Calu6 non-small lung cancer line and the MX1 breast cancer line in nude mice [8, 9, 104, 117]. This is the only TGF-*β* receptor kinase inhibitor that is currently tested in clinical trials.

SD-093 and LY-580276 have been shown to block EMT and tumor cell migration in pancreatic cancer and mouse mammary epithelial cells, respectively [118]. EW-7203, EW-7195, and EW-7197 inhibited Smad/TGF-*β* signaling, cell migration, invasion, and lung metastasis of breast cancer cells in 4T1 and MDA-MB-231 orthotropic xenograft mice and MMTV/cNeu transgenic mice. They inhibited EMT in both TGF-*β* treated breast cancer cells and 4T1 orthotropic xenograft mice. The dose 1.25 mg/kg of EW-7197 increased the survival time of 4T1-Luc and 4T1 breast tumor bearing mice [119]. Preclinical study with EW-7197 was completed. Fang et al. [3] have shown the efficacy of a novel small molecule YR-209, inhibitor of T*β*RI kinase. They have examined the effects of YR-290 on breast cancer cell migration and metastasis* in vitro* (MDA-MB-231 cell line, mouse breast tumor 4T1 cell line, human breast carcinoma cells Hs578T and BT-549, and human keratinocyte cell line HaCaT) and in tumor metastasis mouse models. YR-290 inhibited breast cancer cell migration, invasion, and EMT induced by TGF-*β* in a dose-dependent manner. In three different mouse tumor metastasis models YR-290 preventively and therapeutically blocked breast cancer pulmonary and skeletal metastasis by suppressing the TGF-*β* pathway. Treatment with YR-290 also statistically significantly prolonged the survival of tumor-bearing mice.

Trx-SARA is an example of a peptide aptamer, which reduces the levels of TGF-*β*-induced Smad-2/-3 in complex with Smad-4, and inhibits EMT after TGF-*β* stimulation in breast cancer epithelial cells [8, 120]. So far no clinical trials have been undertaken with peptide aptamers.

## 14. TGF-***β***1 and Cancer Stem-Like Cells (CSCs)

After initial response to chemotherapy many patients have recurrence of drug resistant metastatic disease, especially in triple-negative breast cancers (TNBCs). Some studies revealed that these relapses could be caused by populations of cancer stem-like cells (CSCs) with self-renewing and tumor-initiating capacities. TGF-*β*1 has been shown to increase stem-like properties in human breast cancer cells [121]. Bhola et al. [121] have analyzed RNA expression in matched pairs of primary breast cancer biopsies before and after chemotherapy. Biopsies after chemotherapy displayed increased RNA transcripts of genes associated with CSCs and TGF-*β*1 signaling. Also Shipitsin and colleagues [53] showed that subpopulations with CSC features (CD44+) within breast tumors overexpressed TGF-*β*1 and the T*β*R1. TGF-*β*1 ligands are often enriched in the TNBC tumor microenvironment and can be produced by tumor cells or by tumor-associated stromal and immune cells [94, 121]. These data suggest the possibility that the TGF-*β* pathway is involved in maintenance of CSCs in breast carcinomas. In a study of Bhola et al. [121] in TNBC cell lines (SUM 159, BT549, SUM149, MDA231) and mouse xenografts, the chemotherapeutic drug paclitaxel increased autocrine TGF-*β*1 signaling and IL-8 expression in CSCs, as indicated by mammosphere formation and CSC markers. The T*β*RI kinase inhibitor LY2157299 and SMAD4 siRNA blocked paclitaxel-induced* IL8* transcription and CSCs expansion, as well as paclitaxel-induced SUM159 and BT549 mammosphere formation. Moreover, treatment of TNBC xenografts with LY2157299 prevented reestablishment of tumors after paclitaxel treatment.

Pathways that control stem-cell proliferation are another option for cancer treatment. The canonical Wnt signaling maintains the growth of stem cells. For example in the intestine, the presence of TGF-*β*-signaling and the absence of Wnt signaling in the villus compartment result in rapid cell cycle arrest and differentiation. Thus, Tcf4 (affected by Wnt signaling) and Smad-4 constitute a dominant switch between the proliferative progenitor and the transitional progenitor of differentiated epithelial cell [8].

## 15. Combination Therapy Approaches

Investigators at Genzyme, Inc., have examined the antitumor effects of the pan-TGF*β* neutralizing antibody, 1D11, in combination with various common chemotherapeutics against mammary cancer models. The combination of 1D11 with CDDP Cisplatin resulted in long-term survivors in the 4T1 murine breast cancer cells in experimental bone metastasis assay. More recently, the same investigators demonstrated at least additive dose-dependent effects of 1D11 against several human tumor xenografts (including breast and renal cell) when combined with a variety of cytotoxic agents, including paclitaxel, CDDP, doxorubicin, or CTX (cyclophosphamide). Similarly, scientists at Genentech have shown that the 2G7 potentiates the efficacy of docetaxel in 4T1 spontaneous lung metastasis assays [20].

Bhola et al. [121] determined whether the efficacy of doxorubicin in the inhibition of tumor growth and lung metastasis could be improved by simultaneous treatment with a pyrazole-based T*β*RI kinase inhibitor (Biogen Idec HTS466284, Eli Lilly LY364947). In these studies, murine breast cancer 4T1 cells were inoculated into both inguinal mammary fat pads of Balb/c mice. Results of these experiments indicated that while the T*β*RI inhibitor alone failed to inhibit tumor growth, it significantly enhanced doxorubicin's antitumor activity.

Bandyopadhyay et al. [122] have reported that inhibiting TGF-*β* signaling in mammary epithelial cells using a chemical T*β*R-I kinase inhibitor attenuated ATM (Ataxia Telangiectasia Mutated kinase) autophosphorylation and significantly reduced its kinase activity, while adding back TGF-*β*1 restored functional ATM and downstream DNA damage responses. These studies have discovered a critical link between activation of TGF-*β*1 in the microenvironment and ATM, which directs epithelial cell genotoxic stress responses and, indirectly, tissue integrity.

In the experiments of Seth et al. [123] effects of antagonization of TGF-*β* were combined with the oncolytic effects of an infectious adenoviral vector. An oncolytic adenovirus expressing Fc:T*β*RII was constructed by homologous recombination. MDA-MB-231 and MCF-7 human breast cancer cells were infected, and the transcription of TGF-*β* was inhibited in targeted cells. Direct injection of virus into MDA-MB-231 human breast carcinoma xenografts caused tumor regression in more than 85% of the animals [20, 123].

## 16. Crosstalk between Estrogen- and TGF***β*** Signaling Pathways in Breast Cancer Cells

A number of studies have suggested that estrogen receptor (ER)-negative (ER−) human breast carcinoma lines were relatively more sensitive to growth inhibition by TGF-*β* than ER+ lines [20]. For example, estrogen dependent MCF-7 breast cancer cells were found to be quite sensitive to TGF*β*-mediated growth inhibition, while estrogen-independent MCF-7 sublines were refractory to TGF*β* [20]. Growth of ER-positive MCF-7 cells is stimulated by estradiol as well as by progestins in a dose-dependent manner, and this effect can be blocked by treatment with 4-hydroxy-tamoxifen (4-OH-T). Moreover, estradiol- or norethindrone-induced growth stimulation is accompanied by a dramatic decrease in TGF*β*2 and -3 mRNA levels, whereas the level of TGF*β*1 mRNA was not affected [20].

Manni et al. [124] have demonstrated that even though treatment with TGF-*β* had no effect on MCF-7 cell growth in 2D culture, it inhibited colony formation in 3D in a dose-dependent manner to a degree comparable to that observed with 4-OH-T. Furthermore, the growth inhibitory effect of 4-OH-T was completely reversed by an anti-TGF*β* antibody. These observations suggested that TGF*β* might act on a small TGF*β*-responsive progenitor cell population, at least in 3D cultures of MCF7 cells. These findings are similar to those reported by Shipitsin et al. [53] who have examined gene expression and genetic profiles of cells isolated from cancerous and normal breast tissue using the cell surface markers,* CD44* and* CD24*. Most tumors contained cell populations that were either predominantly ER+,* CD44*−,* CD24*+ or ER−,* CD44*+,* CD24lo*. Moreover, the* TGFBR2* gene was selectively expressed in ER−* CD44*+,* CD24lo* mammary epithelial precursors, but was epigenetically silenced in differentiated, ER+* CD44*−,* CD24*+ luminal cells. Thus, differentiation into luminal cells appeared to be associated with inactivation of TGF*β* signaling.

It is worth noting that tamoxifen-responsiveness* in vivo* may depend not only on ER expression in the breast cancer cells but also on stem cell population sensitivity to TGF*β*-mediated growth arrest. Thus, in some cases, ER+ tumors might become resistant to antiestrogens because the tumor stem cells no longer respond to TGF*β*-mediated cell cycle arrest. In this situation, constitutive inactivation of TGF-*β* signaling may even contribute to antiestrogen resistance [20]. On the basis of several observations the postulate has been created that in the early stages of breast cancer development, the mammary epithelial stem cell population is still sensitive to growth inhibition by TGF-*β* (and, thus, sensitive to tamoxifen if there is an ER+ subpopulation). During breast cancer progression the cells escape from TGF*β*-mediated growth arrest, and the higher levels of TGF-*β* could be associated with greater invasive and/or metastatic potential and tamoxifen-resistance [20].

## 17. Crosstalk between HER2 Kinase and TGF***β*** Signaling in Mammary Tumor Progression

*HER2 *gene amplification is reported in approximately 25% of metastatic breastcancers, where it is associated with poor patient outcome [125]. Studies of HER2-overexpressing breast cancer cell lines and human tumors have shown constitutive HER2 phosphorylation and activation. Overexpression of HER2 is associated with mammary epithelial cell transformation and shorter survival in breast cancer patients [20, 49, 125]. In breast cancer models (*in vitro* and* in vivo*—in mice expressing the Neu oncogene), a functional synergy between TGF-*β* and HER2 has been characterized. Exogenous as well as transduced TGF-*β* confer motility and invasiveness in MCF10A cells (HMECs), which were showing stable expression of transfected HER2 [125, 126]. Such experiments showed that TGF-*β*1 and TGF-*β*3 cDNAs cooperate with HER2 in inducing cell motility and invasion in both 2D and 3D basement membrane cultures. This cooperation between HER2 and TGF-*β* correlates with sustained activation of AKT, ERK, and p38 MAPK and is abolished by pharmacological inhibition of PI3 K, ERK, or p38 MAPK. Indeed, a genetic modifier screen in these cells identified TGF-**β**1 and TGF-**β**3 as molecules that cooperate with HER2 in inducing cell motility and invasion [49, 125]. Evidence suggest that blockage of HER2:TGF-**β** crosstalk may significantly enhance the efficiency of conventional therapies in breast cancer patients with HER2 overexpression [49].

## 18. TGF***β*** Induced Apoptosis in Tumor Suppression

In epithelial, neuronal, and haematopoietic cells, TGF-*β* limits cell proliferation through a coordinated program of cytostatic gene responses. So far, the unique TGF-*β*-induced apoptotic program characteristic for cancer cells is poorly understood [8, 9].* In vitro* studies have shown some Smad-dependent and -independent mechanisms; for example, TGF-*β* increases the expression of death associated protein kinase (DAPK) in HCC cell-lines [126]. Other apoptotic related genes affected by TGF-*β* pathway are DAXX (that normally activates p38MAPK), FAS, and BIM (in gastric cancer cell lines) and GADD45*β* (growth arrest and DNA damage inducible 45 *β*; in hepatocytes) [8, 9, 60]. The final targets in TGF-*β*-induced apoptosis are the proapoptotic caspases and several members of the BCL2 family [7]. Confirmation of the physiological relevance of these candidates awaits experimental proof using* in vivo* model systems.

## 19. TGF***β*** Induced Autophagy in Breast Cancer Cells

Autophagy is a pivotal response of normal and cancer cells to environmental stress and is induced by various stimuli [127, 128]. Otherwise, autophagy has an intrinsic function in tumor suppression [129]. Although autophagy might allow tumor cells to survive under metabolic stress [128], several genetic links have emerged between defects of autophagy and development of cancer. Metastatic cancer cells may escape from anoikis via the induction of autophagy [130, 131].* BECLIN1* is monoallelically deleted in 40% to 75% of human breast, ovarian, and prostate cancers, and thus considered as a tumor suppressor gene [128, 132, 133]. Accordingly, heterozygous deletion of* BECLIN1* in mice (*beclin 1+/−*) resulted in increased incidence of spontaneous tumors [128, 132]. Many breast carcinoma cell lines, although polyploid for chromosome 17 (*beclin1* gene is placed on chromosome 17q21), exhibit deletions of one or more* beclin1* alleles13 and human breast tumors show decreased Beclin1 levels compared to normal adjacent tissue. Restoration of Beclin1 and autophagy in MCF-7 cells is associated with inhibition of MCF7-induced tumorigenesis in nude mice [128].* beclin1+/−* mice do not have increased incidence of mammary tumors but rather are susceptible to lymphomas and carcinomas of the lung and liver after a long latency [134, 135]. Tumors forming in* beclin1+/−* mice express wild-type* beclin1* mRNA and protein, indicating that* beclin1* is a haploinsufficient tumor suppressor.

Autophagy activation by TGF*β*1 is mediated through the Smad and JNK pathways [129]. In the work of Kiyono et al. [132] TGF-*β* treatment induced the formation of GFP-LC3 puncta in human MDA-MB-231 mammary carcinoma cells and in mouse mammary carcinoma cell line, JygMC. Moreover, TGF-*β* enhanced degradation of long-lived proteins in MDA-MB-231 cells. Autophagic cell death has been also described in anti-estrogen-treated cultured human mammary carcinoma MCF-7 cells [22].

The role of autophagy might be different in certain stages and aspects of tumor development. Various tumor suppressors (e.g., PTEN, TSC1/2, p53, and DAPK) are autophagy inducers, whereas some inhibitors of autophagy (e.g., Akt and Ras) possess oncogenic activity [136]. Because TGF-*β* primarily functions as a tumor suppressor in early stages of carcinogenesis, TGF-*β*-induced autophagy may suppress tumor initiation in cooperation with other tumor suppressors. In later stages of tumor progression, it was shown that the metabolically stressed regions of the tumor mass activate autophagy. In this scenario, autophagy activation might confer a growth advantage to these cells. Regarding the tumor-promoting aspects of TGF-*β* in advanced cancer, TGF-*β*-induced autophagy in certain tumor types, including breast cancer, might be implicated in tumor promotion in the later phase of tumorigenesis [132, 137]. In the work of Suzuki et al. [129] TGF*β*-induced autophagy was suppressed by the knockdown of Smad2/3, Smad4, or DAPK, or inhibition of JNK, indicating the involvement of both Smad and non-Smad pathways. TGF*β*1 activates autophagy earlier than execution of apoptosis, and silencing of autophagy genes by siRNA attenuates the cell cycle arrest and apoptosis induction by TGF*β*1 in HuH7 cells (human hepatocellular carcinoma cells), indicating that autophagy activation should partially contribute to TGF*β*-mediated growth inhibition.

Concepts of autophagy inhibition used in cancer therapy have led to trials testing autophagy inhibitors, such as chloroquine, as sensitizers for radio- and chemotherapy in several malignancies [138].

## 20. Concluding Remarks

Direct impact of TGF-*β*1 upon the carcinoma cells, as well as regulation of carcinoma-immune cells interactions by this cytokine, must be considered when designing relevant therapeutic approaches to manage human cancer progression and metastasis. The prognostic utility of TGF-*β*1 in human BC has also been described. Elucidation of the molecular mechanisms responsible for conferring oncogenic activities of TGF-*β*1 will undoubtedly provide new therapeutic opportunities to alleviate metastatic progression and disease recurrence of BC. Currently some anti-TGF-*β*1 therapies are being explored. Given the role of microRNAs in mediating EMT and TGF-*β* signaling, it stands to reason that identifying the micro-RNAome regulated by TGF-*β* during its induction of metastatic progression may also offer new inroads to enhance the overall survival of breast cancer patients.
